# GSK3640254 Is a Novel HIV-1 Maturation Inhibitor with an Optimized Virology Profile

**DOI:** 10.1128/AAC.01876-21

**Published:** 2022-01-18

**Authors:** Ira Dicker, Jerry L. Jeffrey, Tricia Protack, Zeyu Lin, Mark Cockett, Yan Chen, Sing-Yuen Sit, Martin Gartland, Nicholas A. Meanwell, Alicia Regueiro-Ren, Dieter Drexler, Joseph Cantone, Brian McAuliffe, Mark Krystal

**Affiliations:** a ViiV Healthcare, Branford, Connecticut, USA; b ViiV Healthcare, Research Triangle Park, North Carolina, USA; c Bristol Myers Squibb, Wallingford, Connecticut, USA

**Keywords:** maturation inhibitors, HIV, novel therapeutics, GSK3640254, resistance profile

## Abstract

HIV-1 maturation inhibitors (MIs) offer a novel mechanism of action and potential for use in HIV-1 treatment. Prior MIs displayed clinical efficacy but were associated with the emergence of resistance and some gastrointestinal tolerability events. Treatment with the potentially safer next-generation MI GSK3640254 (GSK’254) resulted in up to a 2-log_10_ viral load reduction in a phase IIa proof-of-concept study. *In vitro* experiments have defined the antiviral and resistance profiles for GSK’254. The compound displayed strong antiviral activity against a library of subtype B and C chimeric viruses containing Gag polymorphisms and site-directed mutants previously shown to affect potency of earlier-generation MIs, with a mean protein-binding adjusted 90% effective concentration (EC_90_) of 33 nM. Furthermore, GSK’254 exhibited robust antiviral activity against a panel of HIV-1 clinical isolates, with a mean EC_50_ of 9 nM. Mechanistic studies established that bound GSK’254 dissociated on average 7.1-fold more slowly from wild-type Gag virus-like particles (VLPs) than a previous-generation MI. In resistance studies, the previously identified A364V Gag region mutation was selected under MI pressure in cell culture and during the phase IIa clinical study. As expected, GSK’254 inhibited cleavage of p25 in a range of polymorphic HIV-1 Gag VLPs. Virus-like particles containing the A364V mutation exhibited a p25 cleavage rate 9.3 times higher than wild-type particles, providing a possible mechanism for MI resistance. The findings demonstrate that GSK’254 potently inhibits a broad range of HIV-1 strains expressing Gag polymorphisms.

## INTRODUCTION

Even with the success of modern first-line therapies, current challenges for people infected with HIV-1 include the development or direct transmission of drug resistance (1). Thus, the development of antiretroviral therapy agents with novel mechanisms of action, particularly for people with HIV-1 who have experienced virologic failure, is needed. Ideal therapies will exhibit robust HIV-1 antiviral activity against a broad range of HIV-1 subtypes without cross-resistance to currently used therapies.

Current anti-HIV combination therapy targets multiple steps of the HIV-1 life cycle (2). Maturation inhibitors (MIs) offer a novel mechanism of action by blocking a late step in the virus life cycle to prevent the creation of infectious virions (3). Specifically, MIs block the cleavage of p25 into the p24 capsid protein and spacer peptide 1 (SP1), a step that normally permits protein restructuring to allow for development of mature infectious virions (3, 4). In a phase IIa study, the MI bevirimat was efficacious; however, it was ineffective against viruses containing Gag polymorphisms such as V362I and other fairly common polymorphisms between amino acids 369 through 371 of the Gag protein (5–9). Subsequent *in vitro* studies of a previous MI, GSK3532795 (GSK’795), found that it exhibited improved potency compared with that of bevirimat against viruses containing these polymorphisms (10). However, in a phase IIa study assessing GSK’795 monotherapy, participants with HIV-1 containing certain more complex Gag polymorphisms at baseline, such as V362I/V370A, displayed reduced antiviral sensitivity to GSK’795 (11, 12). Over 10-day treatment with GSK’795 monotherapy ranging from 10 to 120 mg/kg of body weight, Gag polymorphisms were again detected that conferred reduced susceptibility to GSK’795, and an A364V mutation which was previously observed to confer resistance to the MI bevirimat was emergent (7, 11). During the phase IIb study, GSK’795 in combination with the nucleoside reverse transcriptase inhibitors (NRTIs) emtricitabine and tenofovir disoproxil fumarate elicited a number of gastrointestinal adverse events, resulting in the discontinuation of GSK’795 (13).

**FIG 1:**
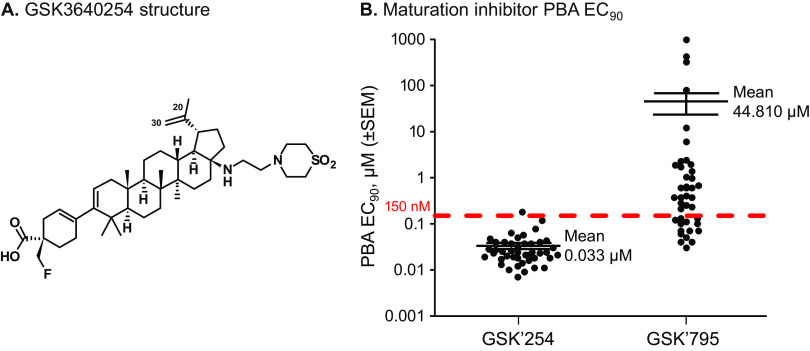
Structure of GSK’254 and MI EC_90_ against a minilibrary of clinically relevant HIV-1 polymorphisms in a multiple-cycle assay. (A) Structure of GSK’254. The C-20/C-30 double bond that was reduced to make radiolabeled surrogate maturation inhibitors is shown. (B) PBA EC_90_ of GSK’254 and GSK’795 against a minilibrary of HIV-1 polymorphisms. The estimated *C*_min_ for GSK’254 was 150 nM, as represented by the red dashed line. Mean values and standard errors of the means are graphed for each MI. The mean value for GSK’795 was calculated using 3 fewer samples, as 3 viruses did not have a measurable EC_90_ (V362I/V370A- and A326T/V362I/V370A-containing viruses and a virus containing the 93BR024 gag/pro gene). *C*_min_, steady-state serum concentration; EC_90_, 90% effective concentration; GSK’254, GSK3640254; GSK’795, GSK3532795; MI, maturation inhibitor; PBA, protein-binding adjusted.

The next-generation MI GSK3640254 (GSK’254) (Fig. 1A) was developed through a medicinal chemistry approach coupled with a virology triage strategy focused on improving the safety profile and spectrum by targeting coverage of key Gag polymorphisms that typically impact susceptibility to MIs (14). Phase I trials found that oral GSK’254 taken alone, or in combination with tenofovir alafenamide/emtricitabine or dolutegravir, displayed acceptable safety and tolerability in healthy participants, supporting the clinical potential of GSK’254 (15, 16). In a phase IIa proof-of-concept study, 10-day monotherapy with 200 mg of GSK’254 daily exhibited up to a 2.01-log_10_ viral load reduction. However, the A364A/V mutation emerged in 4 of the 6 participants receiving GSK’254 at this dose by day 11, with 1 of the 4 participants also exhibiting phenotypic resistance (17). As a result of this, additional groups in the study were dosed with GSK’254 for 7 days, and no further emergent A364V was observed. Gag polymorphisms at baseline in this cohort did not seem to impact GSK’254 antiviral activity.

In this study, the antiviral activity and mechanism of action of GSK’254 were characterized against a range of HIV-1 subtypes (both clinical isolates and chimeric viruses) and Gag polymorphisms *in vitro*. Furthermore, the antiviral activity of GSK’254 was compared with that of the previous-generation MI GSK’795 (10–12).

## RESULTS

### GSK’254 exhibits antiviral activity against genotyped subtype B and C viruses in a multiple-cycle replication assay.

Using a multiple-cycle replication assay, GSK’254 was screened against a genotypically diverse panel of NLRepRluc viruses containing *gag/pr* genes derived from subtype B or subtype C viruses or NLRepRlucP373S viruses containing Gag site-directed mutants (SDMs). Aggregated activity against the phenotyped panel of subtype B and C viruses is shown in Table 1, and individual susceptibility values, including for the SDM viruses, are listed in Table S1 in the supplemental material. GSK’254 exhibited excellent activity against all tested viruses, with mean half-maximal effective concentration (EC_50_) values against subtype B and C *gag/pr*-containing viruses of 1.9 and 1.2 nM, respectively. Viruses were highly susceptible to GSK’254, with the least susceptible virus exhibiting an EC_50_ of 7 nM. Compared with the earlier MI, GSK’795, GSK’254 exhibited superior antiviral activity across this cohort. Viruses with V362I were approximately 4-fold more sensitive to GSK’254 than to GSK’795, whereas the one chimeric subtype B virus containing V362I/V370A was much more sensitive to GSK’254. For the panel of 35 phenotyped viruses, 32/35 were more susceptible to GSK’254 than to GSK’795, with 6/35 viruses exhibiting a 10-fold or greater difference between the 2 compounds and 3/35 viruses having equivalent susceptibilities to both MIs.

**TABLE 1:** Antiviral activity against Gag/Pr genotyped subtype B and C viruses in a multiple-cycle assay^*a*^

Virus type	*n*	GSK’254		GSK’795	
		Mean ± SD EC_50_, nM	Median (range) EC_50_, nM	Mean ± SD EC_50_, nM	Median (range) EC_50_, nM
Subtype B	24	1.9 ± 1.5	1 (0.5–7)	66.0 ± 284.4	5 (1–1,400)
Subtype C	11	1.2 ± 0.4	1 (1–2)	9.1 ± 5.6	7 (3–22)
Wild-type subtype B^*b*^	5	1.1 ± 0.5	1 (0.5–2)	2.8 ± 2.5	1 (1–6)
V362I only	9	2.7 ± 2.0	2 (1–7)	11.1 ± 16.3	5 (3–54)
V362I/ΔV370	1	3		1,400	

a EC_50_, half-maximal effective concentration; GSK’254, GSK3640254; GSK’795, GSK3532795.

b Wild type defined as having V362, Q369, V370, and T371.

To determine the human serum effect on GSK’254, antiviral activity of the RepRlucP373S virus was assessed in the presence of 40% human serum supplemented with 27 mg/mL of human serum albumin to reach an albumin concentration equivalent to that in 100% human serum. Three different polymorphism-containing viruses were used: the wild type (WT), V370A-containing virus, and a ΔV370-containing virus. As expected, the 3 viruses exhibited similar serum effects under these conditions, with GSK’254 potency decreasing an average of 7.9-fold (Table 2). This trend corresponds well to the measurement of human protein binding determined for GSK’254 in an ultracentrifugation assay, which was 86% ± 2%. Furthermore, GSK’254 cytotoxicity was assessed in MT-2 cells, and the compound exhibited a half-maximal cytotoxic concentration (CC_50_) of 13 ± 6 μM, resulting in a therapeutic index well over 1,000 for all viruses shown in Table 1.

**TABLE 2:** Potency of GSK’254 in the presence of serum in multiple-cycle assay^*a*^

Virus	10% FBS			10% FBS + 40% HS + 27 mg/mL of HSA^*b*^			Serum effect (±HS EC_50_ ratio)
	EC_50_, nM	EC_90_, nM	*n*	EC_50_, nM	EC_90_, nM	*n*	
Wild type	1.4	3.6	58	12.3	26.1	40	8.8
V370A	1.5	3.0	4	9.1	42.2	4	6.1
ΔV370	4.4	7.8	6	38.2	195	6	8.7

a Assays used luciferase endpoint. FBS, fetal bovine serum; HS, human serum; HSA, human serum albumin.

b Albumin concentration equivalent to 100% human serum.

### Estimation of GSK’254 minimum blood plasma concentration required for clinical efficacy.

The antiviral activities of GSK’795 and GSK’254 against a 44-member library of genotyped chimeric viruses and SDM viruses (the panel of 35 phenotyped viruses plus 9 SDM-containing viruses) containing Gag polymorphisms are graphed in Fig. 1B, with the genotypes, EC_50_, and protein-binding adjusted (PBA) 90% effective concentration (PBA EC_90_) values listed in Table S1. These viruses were chosen to be representative of the population of Gag genes as a whole, including known polymorphism-containing viruses with reduced susceptibility to GSK’795, as well as SDM viruses with multiple changes that had previously been identified as relevant for susceptibility to earlier MIs (7, 11). GSK’254 had a mean PBA EC_90_ of 33 nM against this cohort, while the mean PBA EC_90_ value for GSK’795 was at least 1,624-fold higher (not counting the 3 viruses for which an EC_90_ was not attainable with GSK’795). It was estimated that a human steady-state serum concentration (*C*_min_) of 150 nM would be multiples higher than the PBA EC_90_ values of the vast majority of HIV-1 isolates and provided a target *C*_min_ for clinical studies.

### Antiviral activity against laboratory strains.

GSK’254 antiviral activity against 8 HIV-1 laboratory strains and 2 HIV-2 strains is presented in Table 3. Virus yields were measured in dose-response experiments by either luciferase activity in human T-cells (CEM-NKR-CCR5-Luc), extracellular reverse transcriptase activity in MT-2 cells (T-tropic viruses), or extracellular p24 antigen in PM1 cells (M-tropic viruses). GSK’254 EC_50_ values determined using the reporter cell line were less than 1 nM for T-tropic HIV-1, and similar values were obtained for M-tropic HIV-1 Bal (1.1 nM) and JRFL (1.2 nM) using p24 antigen as the endpoint. The mean (standard error of the mean) EC_50_ value of GSK’254 against these 8 HIV-1 laboratory strains was 0.8 (±0.10 nM), with a range of 0.3 to 1.2 nM. GSK’254 and GSK’795 were active against the ROD laboratory-adapted HIV-2 strain in MT-2 cells using a reverse transcriptase endpoint but was much less active in the CEM-NKR-CCR5-Luc cells using a luciferase endpoint. The reason for this difference is unclear. Weak activity was observed for both compounds against the HIV-2 287 strain.

**TABLE 3:** GSK’254 activity against laboratory strains

Virus	GSK’254 EC_50_ ± SD, nM^*a*^	GSK’795 EC_50_ ± SD, nM^*b*^
HIV-1 T tropic		
RF	0.9 ± 0.15	6.9 ± 2.8
IIIB	0.6 ± 0.40	2.5 ± 0.8
HXB2	0.7 ± 0.10	9.3 ± 3.9
NL_4-3_	0.9 ± 0.20, 7.3^*b*^	3.9 ± 2.2, 19
LAI	0.7 ± 0.10	2.4 ± 0.8
MN	0.3	1.0
HIV-1 M tropic		
Bal	1.1^*c*^	3.7^*c*^
JRFL	1.2^*c*^	13^*c*^
HIV-2		
ROD	>3,000, 2.5^*b*^	>3,000, 17
287	1,245, >1,386^*b*^	751

a Activity determined through inhibition of luciferase activity in CEM-NKR-CCR5-Luc cells unless otherwise noted.

b Activity determined through inhibition of reverse transcriptase activity in MT-2 cells unless otherwise noted.

c Activity determined through inhibition of p24 in PM1 cells.

### GSK’254 exhibits robust antiviral activity against clinical isolates in PBMCs.

The antiviral activity of GSK’254 was also evaluated using a panel of HIV-1 clinical isolates representing 4 subtypes: A (*n* = 3), B (*n* = 7), C (*n* = 6), and CRF01 (AE; *n* = 3) (Table 4). Human peripheral blood mononuclear cells (PBMCs) were infected with isolates, and virus yields were measured by determining extracellular p24 protein levels (Table 4). Overall, GSK’254 displayed higher potency toward clinical isolates than GSK’795, with mean EC_50_ values of 9 versus 138 nM, respectively. Activity was similar among C and A/AE subtypes, with mean values of 3.5 and 3.0 nM, respectively. The mean EC_50_ of GSK’254 toward subtype B was higher (19 nM) due to reduced activity observed against 2 subtype B isolates, 93BR008 and 92BR018, with EC_50_ values of 50 and 77 nM, respectively. These isolates were also examined in the NLRepRlucP373S virus by replacement of the *gag/pr* genes with those from these clinical isolates. Interestingly, 93BR008- and 92BR018-genotyped viruses exhibited values of 2 and 1 nM, respectively, in multiple-cycle assays (Table S1). This suggests that these viruses are likely susceptible to GSK’254, with potency differences in the 2 formats likely due to differences in multiplicity of infection (MOI) or other factors in the PBMC-based assay.

**TABLE 4:** Inhibition of clinical isolates in PBMCs by MIs

Subtype	Virus name	Gag polymorphism(s)^*a*^	Mean EC_50_, μM	
			GSK’254	GSK’795
B	93BR009	V218A/V370M/N372G	0.001	0.002
B	92BR003	A374P/T375N	0.002	0.004
B	93US143	I376M	0.002	ND^*b*^
B	93BR013	V370I/T375N	0.003	0.009
B	93BR008	V362I/N372T/A374T/T375N	0.050	0.123
B	92BR018	V218A/H219Q/V370A/ΔT371/T375A	0.077	0.145
B	93BR017	V218A/H219Q/A326S/V370A/ΔT371/S373T/T375A	0.001	0.010
C	98BR004	V218A/H219Q/A326S/ΔT371/A374T/T375N	0.001	0.001
C	MJ4	V218I/R286K/V370A/ΔT371/A374T/T375S	0.003	0.007
C	20706-3	ΔV370/A374S/ΔT375	0.004	0.004
C	93MW960	R286K/V370A/ΔlT371/ΔA374/T375N	0.009	1.575
C	10215-6	H219Q/R286K/ΔV370A/ΔT375	0.001	0.013
C	97ZA012	Not sequenced	0.003	0.004
A	93RW029	V218A/H219Q/T332S/P339T/A340G/G357S/V362I/V370A/T371Q/S373T/A374N	0.003	0.006
A	93RW034	Not sequenced	0.001	0.002
A	92UG037	Not sequenced	0.005	0.002
AE	93TH053	T332S/Q369F/V370A/ΔT371/N372Q/S373Q/T375N	0.005	0.018
AE	93TH023	T332S/V370A/ΔT371/N372Q/S373N/A374V/T375N	0.001	0.533
AE	CMU010	T332S/V370A/ΔT371/N372Q/S373H/T375N	0.003	0.035

a Defined by changes at positions identified during resistance selection with GSK’795 (11) and changes from consensus between amino acids 369 and 376.

b ND, not determined.

### Effect of site-directed substitutions on GSK’254 susceptibility in multiple-cycle and single-cycle assays.

An extensive analysis of GSK’254 toward viruses with reduced susceptibility to GSK’795 was performed in single-cycle and multiple-cycle replication assays against viruses containing Gag region SDMs (Table 5). GSK’254 exhibited greater potency than did GSK’795, as evidenced by lower EC_50_ values and higher values for maximal percent inhibition (MPI) in both single- and multiple-cycle assays. Notably, viruses expressing Gag polymorphisms at V370 have been shown to exhibit reduced susceptibility to bevirimat (7), while GSK’254 suppresses most SDMs with mutations located at V370 in single- and multiple-cycle assays except for those containing A366V/V370M. The highly GSK’795-resistant R361K/V362I/L363M triple variant was previously observed *in vivo* (18) but was inhibited by GSK’254 with EC_50_ values of 14 nM (MPI, 89%) in the single-cycle assay and 3 nM (MPI, 91%) in the multiple-cycle assay. The T332S/V362I/PrR41G genotype was previously reported as selected in the presence of GSK’795 in a sequential-passage experiment and promoted high-level GSK’795 resistance (11). Against GSK’254, T332S/V362I/PrR41G exhibited EC_50_ values of 13 nM (MPI, 88%) in the single-cycle assay and 7 nM (MPI, 97%) in the multiple-cycle assay (11). Viruses containing A366V or A364V substitutions generally exhibited greatly reduced susceptibility to GSK’254. Notably, A366V is not present in the Los Alamos National Laboratory (LANL) HIV sequence database. Altogether, these results show that common Gag polymorphisms as well as complex *in vivo* and GSK’795-selected resistance variants generally exhibited low EC_50_ values and high MPI values for GSK’254.

**TABLE 5:** Antiviral activity of GSK’254 against SDMs in single-cycle and multiple-cycle assays

Assay type and virus	GSK’254			GSK’795		
	Mean ± SD EC_50_, μM	FC-EC_50_	MPI, %	Mean ± SD EC_50_, μM	FC-EC_50_	MPI, %
Single-cycle assay						
Wild type	0.003 ± 0.002	1.0	98	0.005 ± 0.003	1.0	94
V370A	0.002 ± 0.001	0.7	97	0.006 ± 0.000	1.2	84
V370M	0.002 ± 0.001	0.7	93	0.013 ± 0.011	2.6	78
ΔV370	0.008	2.7	96	0.110	2.2	60
T33N/V370A	0.003	1.0	94	0.030	6.0	70
V362I	0.003 ± 0.001	1.0	89	0.014 ± 0.006	2.8	68
M367V	0.015 ± 0.010	5.0	89	>3	>600	36
V362I/V370A	0.004 ± 0.001	1.3	91	>3	>600	31
V362I/V370M	0.004 ± 0.002	1.3	90	>3	>600	51
V362I/H219Q	0.007 ± 0.002	2.3	91	0.066	13.2	60
H358Y/ΔT371	0.035 ± 0.002	11.7	67	>3	>600	18
I333V/V370A	0.011 ± 0.001	3.7	86	>3	>600	10
I333V/V370	0.011 ± 0.001	3.7	86	>3	>600	−36
I333V/V362I	0.039 ± 0.005	13.0	65	>3	>600	−30
T332N/V370A	0.004 ± 0.001	2.2	94	0.030	7.6	70
T332S/V362I/prR41G	0.013 ± 0.009	4.3	88	>3	>600	−30
V362I/H219Q/T332S	0.008 ± 0.003	2.7	91	0.71	142	52
R361K/V362I/L363M	0.014 ± 0.002	4.7	89	>3	>600	36
A364V	>3	>1,000	45	>3	>600	8
A366V	>3	>1,000	−7.6	>3	>600	3
V362I/A366V	>3	>1,000	−96	>3	>600	−109
A366V/V370M	>3	>1,000	26	>3	>600	3
V362I/A366V/V370M	>3	>1,000	1	>3	>600	−62
Multiple-cycle assay						
Wild type	0.003 ± 0.002	1.0	98	0.005 ± 0.003	1.0	94
V370A	0.002 ± 0.001	0.7	97	0.006	1.2	84
ΔV370	0.003 ± 0.001	1.0	96	0.006 ± 0.002	1.2	87
V362I/V370A	0.003 ± 0.001	1.0	91	>3	>600	31
T332S/V362I/prR41G	0.007 ± 0.004	2.3	97	0.704 ± 0.606	141	65
A326T/V362I/V370A	0.026 ± 0.070	8.7	69	>3	>600	6
R361K/V362I/L363M	0.003 ± 0.001	1.0	91	0.552 ± 0.971	110.4	68
A364V	0.274 ± 0.505	91	70	>3	>600	16
A366V-containing viruses^*a*^	>3	>1,000	−86 to 400	>3	>600	−13 to 408

a Includes genotypes A366V, V362I/A366V, V362I/A366V/V370M, and A366V/V370M.

### Virus breakthrough studies to examine resistance barrier.

The ability of virus to break through and grow in the presence of an inhibitory concentration of a compound in cell culture provides an indication of its resistance barrier. The breakthrough of virus growth as measured by appearance of viral cytopathic effect (CPE) in the presence of GSK’254 was assessed in comparison with that of GSK’795. Experiments were performed in duplicate at a low or high MOI (0.005 and 0.05) and at a drug concentration of 80 nM with RepRlucP373S WT virus or SDM-containing virus with known Gag polymorphisms (Table 6). Across both low and high MOIs, GSK’254 exhibited a greater resistance barrier than GSK’795, as 9 of 16 samples exhibited breakthrough in the presence of GSK’254 versus 15 of 16 in the presence of GSK’795 over the 46-day experiment. Also, in most samples that did exhibit breakthrough, a greater latency to breakthrough was observed with GSK’254 than with GSK’795 (Table 6).

**TABLE 6:** Days to virus breakthrough under MI pressure with low and high MOIs

MOI and virus^*a*^	GSK’254^*b*^		GSK’795^*b*^	
	Expt 1, days^*c*^	Expt 2, days	Expt 1, days	Expt 2, days
Low MOI (0.005)				
Wild type	14 (A366V)	>46	>46	14 (A366A/V)
V370A	>46	11 (A364V)	11 (V362I)	18
V362I	46 (P289P/S)	>46	7	11
V370M	>46	18 (A364V)	7	11
High MOI (0.05)				
Wild type	>46	18 (A364A/V, A366A/V)	11 (A366A/V)	14 (A366A/V, V362V/I)
V370A	>46	>46	11	11
V362I	11 (A364V)	11 (A364V)	7	7
V370M	21 (A366A/V)	11 (A366V)	7	7

a All viruses expressed *Renilla* luciferase reporter.

b Experiments were run in duplicate. The drug was used at 80 nM.

c Selected Gag region mutations reported in parentheses.

Analysis of the Gag genotype in viruses that exhibited breakthrough generally found that GSK’254 treatment selected for virus containing the A364V or A364A/V change (5 of 9 samples with genotypic changes), with 2 instances each of the A366V and A366A/V substitutions observed. A P289P/S substitution was also observed in 1 breakthrough, although the effect of this change on GSK’254 susceptibility is not known.

### Selection of virus with decreased susceptibility to GSK’254 through serial passage.

**FIG 2:**
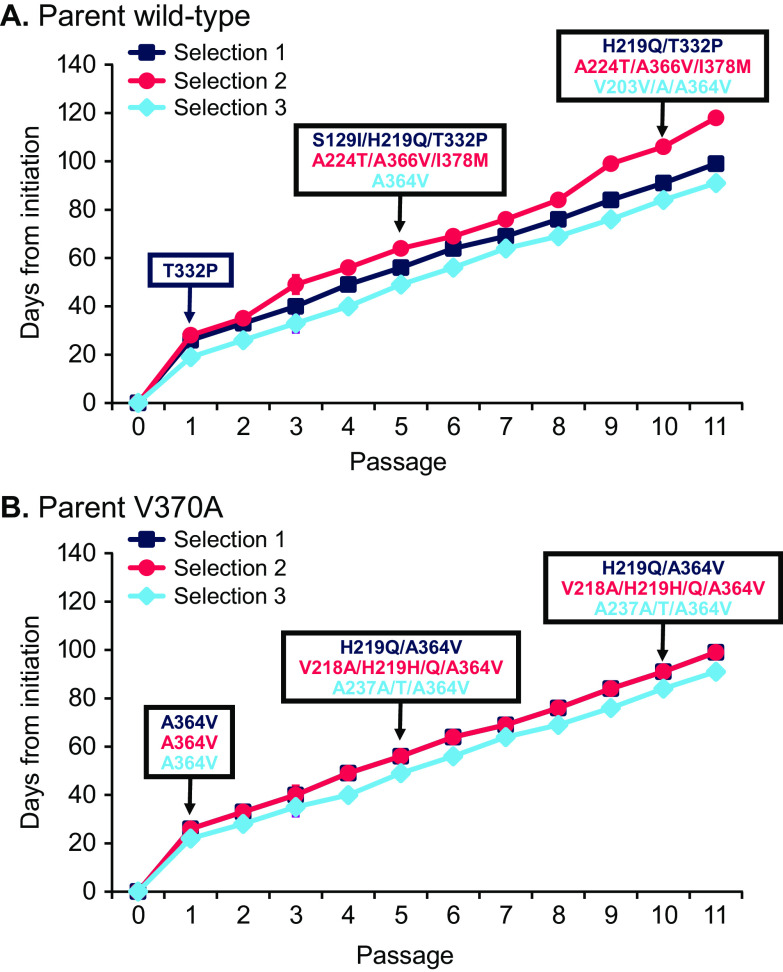
Emergence of mutations during selection with GSK’254 in a multipassage design. Virus was exposed to increasing GSK’254 concentrations originating from parent wild type and V370A variants, and viral genotypes were assessed. (A) Parent wild-type HIV. (B) Parent V370A.

Two NL_4-3_P373S viruses (WT and V370A-containing virus with no luciferase marker present) were used in serial-passage studies to select for virus with reduced susceptibility to GSK’254. For both viruses, replication was dramatically diminished in the presence of the starting concentration of 2 nM GSK’254 (Fig. 2). The average time until there was a sufficient viral titer to permit passage 1 was 24 days, with some culture-to-culture variations.

Resistance development in the WT virus took 3 independent paths (Fig. 2A). T332P was identified in selection 1 at passage 1. Interestingly, T332S was observed in resistance selection by GSK’795, together with V362I (11). No changes were detected in the other 2 WT cultures at passage 1. In selection 1, T332P was maintained but acquired H219Q at passage 5 through passage 10. Notably, the H219Q substitution was also observed in selection experiments with GSK’795 (11) and is a substitution likely involved in cyclophilin recruitment *in vitro* (19–21). S129I was also observed at passage 5 in selection 1 but was absent at passage 10. Selection 2 acquired A366V coselected with A224T and I378M, present at both passage 5 and passage 10. Selection 3 acquired A364V at passage 5, with an additional V203V/A change observed at passage 10.

A364V was the predominant substitution selected for in the parent V370A background. All 3 cultures selected for A364V by passage 1, and it was maintained in subsequent passages (Fig. 2B). At passage 5, additional mutations were variably acquired in the 3 selections and maintained through passage 10. H219Q/H was present in selections 1 and 2, and V218A was coselected with H219Q in selection 2. Selection 3 acquired A237A/T along with A364V.

### GSK’254 mechanism of action.

**FIG 3:**
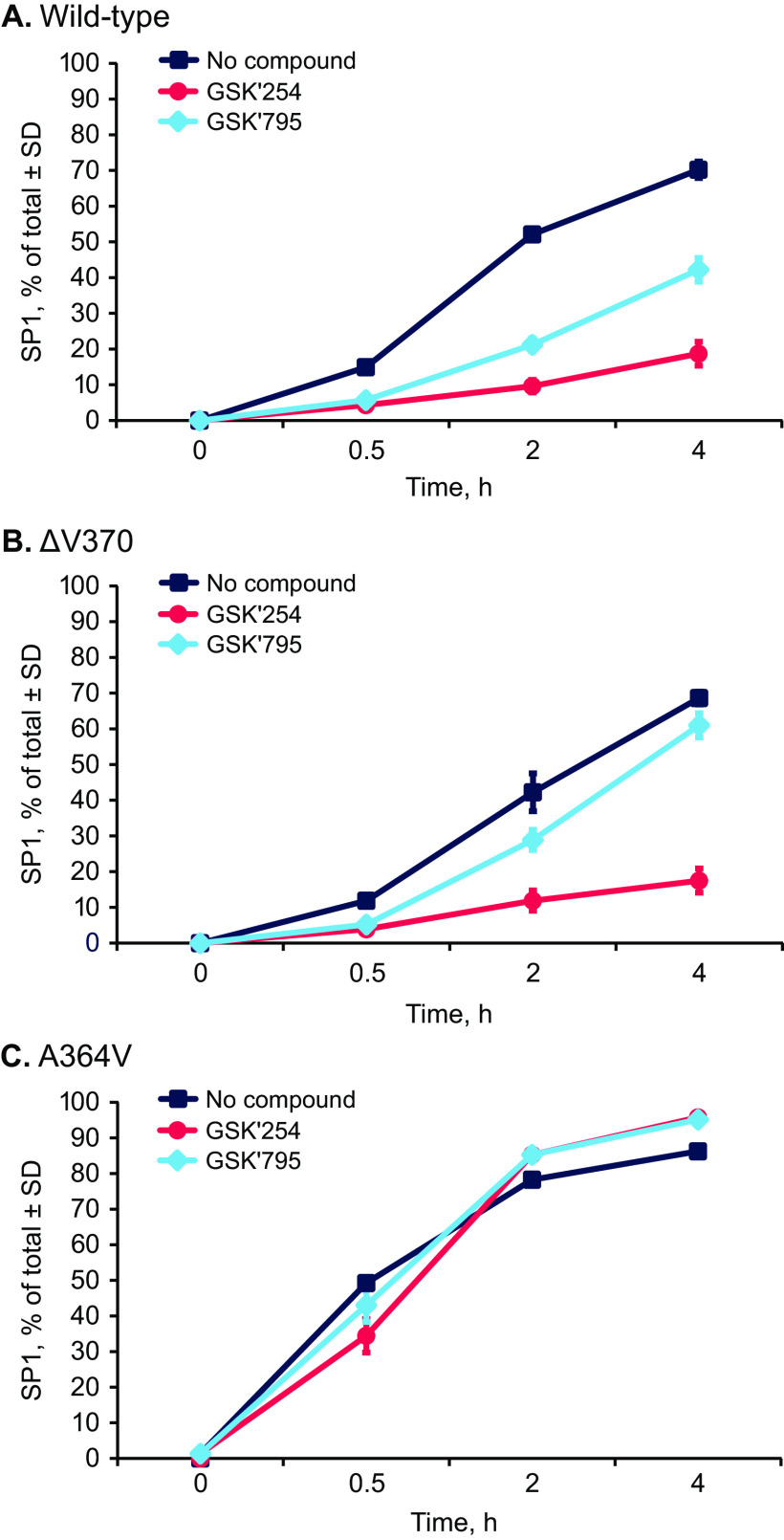
Inhibition of Gag cleavage as assessed by liquid chromatography and mass spectrometry. Normalized SP1 was measured over 4 h in the presence of GSK’254 and GSK’795. (A) Wild-type VLP. (B) ΔV370-containing VLP. (C) A364V-containing VLP. SP1, spacer peptide 1; VLP, virus-like protein.

Akin to previously described MIs, the mechanism of action of GSK’254 is presumed to be inhibition of the last step of protease cleavage of Gag, forming p24 from p25. To demonstrate this, delipidated VLPs were incubated with HIV-1 protease, and the ability of GSK’254 to inhibit p25 cleavage was assessed *in vitro* using liquid chromatography with mass spectrometry analysis to measure loss of p25 as it is converted to p24 and SP1, as has previously been reported for GSK’795 (22, 23). Virus-like particles of 3 genotypes were constructed and tested. In addition to WT VLP, a VLP with V370 deleted (ΔV370; a genotype characteristic of subtype C viruses) and one with the known resistance substitution A364V were analyzed. GSK’254 exhibited significantly better inhibition of p25 cleavage than GSK’795 toward both WT and ΔV370 VLPs (Fig. 3A and B). A364V is a substitution adjacent to the p25/p24 cleavage site (L363/A364), and in this assay (Fig. 3C), neither MI inhibited production of p24 in the presence of HIV-1 protease.

These 3 VLPs, along with 3 additional VLPs containing Gag polymorphisms (V370A/ΔT371, V362I, and V370A), were examined for their innate rates of cleavage by HIV-1 protease in the absence of an MI (22). As shown in Table 7, the first-order rate constant, *K*_clv_, for cleavage of WT VLP at room temperature with 270 nM HIV-1 protease was 0.0017 min^−1^, providing a cleavage half-life of 418 min. The VLPs containing the V362I or V370A polymorph were cleaved approximately 3-fold faster than the wild-type VLP, and VLPs containing V370A/ΔT371 or ΔV370 were cleaved 1.8- and 2.6-fold faster than WT VLP. Notably, the A364V-containing VLP was cleaved 9.2-fold faster than WT VLP, as previously reported (22).

**TABLE 7:** Rates of HIV-1 protease cleavage of p25, half-lives of dissociation of MIs, and MI affinities from Gag polymorphisms

Virus or VLP	Innate p25 cleavage		GSK’254 relative cleavage rates vs wild-type	Single-cycle antiviral assay, FCEC_50_ vs wild-type		MI dissociation from HIV-1 VLPs,^*a*^ half-life ± SD, min		Relative GSK’254/GSK’795 dissociative half-lives	MI affinity to HIV-1 VLPs,^*a*^ *K_d_* ± SD, nM		Relative GSK’254/GSK’795 affinity
	*K*_clv_ min^−1^ ± SD	Half-life, min		GSK’254	GSK’795	GSK’254	GSK’795		GSK’254 ± SD	GSK’795 ± SD	
Wild type	0.0017 ± 0.0003	418	1.0	1	1	361 ± 65	51 ± 18	7.1	2.3 ± 2.3	4.8 ± 1.5	2.1
V370A/ΔT371	0.0030 ± 0.0003	235	1.8	ND	ND	459 ± 155	48 ± 17	9.6	ND	26	ND
ΔV370	0.0044 ± 0.0007	158	2.6	2.7	2.2	315 ± 125	46 ± 13	6.8	3.0 ± 3.1	27 ± 17	9.0
V362I	0.0047 ± 0.0005	148	2.8	1.0	2.8	269 ± 89	45 ± 17	6.0	0.6 ± 0.4	4.3 ± 2.7	7.2
V370A	0.0046 ± 0.0011	156	2.7	0.7	1.2	434 ± 139	36 ± 16	12.1	0.7 ± 0.3	6.5 ± 3.7	9.3
V362I/V370A	0.0097 ± 0.0022	73	5.7	ND	ND	227 ± 83	35 ± 13	6.5	0.5 ± 0.5	5.0 ± 3.6	10.0
A364V	0.0157 ± 0.0013	44	9.2	>1000	>600	≤1	≤1	1.0	15 ± 1	27 ± 10	1.8

a Tritium-labeled GSK’254 and GSK’795 surrogates with the C-20/C-29 double bond reduced with ^3^[H] were used to determine MI dissociation half-life and binding affinity.

Maturation inhibitor dissociation from these VLPs was next determined using a displacement assay with surrogate radiolabeled analogs for GSK’254 and GSK’795 (Table 7). In all cases except for the VLP containing A364V, the GSK’254 surrogate dissociated more slowly than the GSK’795 surrogate from these HIV-1 Gag VLPs (mean GSK’254 surrogate half-life, 344 ± 91 min; mean GSK’795 surrogate half-life, 43 ± 7 min). The GSK’254 surrogate dissociated 7.1-fold more slowly against the WT VLP than the GSK’795 surrogate. Overall, across 6 genotypes (excluding A364V), the GSK’254 surrogate dissociated 8.0-fold (the mean of GSK’254/GSK’795 relative dissociation from HIV-1 VLPs) more slowly than the GSK’795 surrogate. Of note, both compounds dissociated rapidly from A364 VLPs (≤1 min).

The average affinity (dissociation constant [*K_d_*] value) of GSK’254 and GSK’795 surrogate analog binding to these VLPs was then determined. The GSK’254 surrogate had improved binding affinity for these VLPs (mean GSK’254 *K_d_*, 1.4 ± 1.2 nM; mean GSK’795 surrogate *K_d_*, 12.3 ± 11.1 nM [Table 7]). Where data were available for both MIs, the GSK’254 surrogate was found to bind with 7.5-fold-greater affinity (the mean of GSK’254/GSK’795 relative binding affinity toward HIV-1 VLPs, excluding A364V), consistent with its improved antiviral activities toward the cognate viruses. Taken together, improvements in antiviral activity by GSK’254 versus GSK’795 are correlated with improved binding affinity to the variants, which is likely a direct consequence of slower dissociation (longer residence times).

## DISCUSSION

This study examined the *in vitro* antiviral activity and mechanism of action of GSK’254. GSK’254 displayed robust antiviral activity against a broad range of viruses with Gag polymorphisms, including those shown to exhibit reduced susceptibility to previous MIs. However, the A364V substitution was shown to confer reduced susceptibility to GSK’254 in single-cycle and multiple-cycle assays and was acquired in parent HIV-1 strains in selection assays. *In vitro*, GSK’254 inhibited p25 cleavage in WT virus but not virus expressing A364V. While the surrogate analog for GSK’254 dissociated slowly from a panel of polymorphic HIV-1 Gag VLPs (mean half-life, 344 ± 91 min), it dissociated rapidly from the A364V VLP. Altogether, the results from this study demonstrate that GSK’254 has optimized antiviral properties against HIV-1 strains with MI-related Gag polymorphisms but that the A364V mutation remains less susceptible to GSK’254.

GSK’254 was tested against a minilibrary of 35 HIV-1 isolates representative of the Gag diversity in the SP1 region, along with SDMs that were known to have reduced susceptibility to previous generations of MIs (11), including a highly GSK’795-resistant virus identified *in vivo* with the Gag genotype R361K/V362I/L363M (18) (Table 5 and Table S1). Across this cohort, GSK’254 displayed excellent antiviral potency. Based upon effects of human serum on GSK’254 potency, it was estimated that a plasma *C*_min_ of 150 nM for GSK’254 would be higher than multiples of the PBA EC_90_ of almost all HIV-1 isolates. Furthermore, GSK’254 exhibited a greater degree of potency across the minilibrary than GSK’795, a finding that was generally consistent across all experiments comparing the 2 MIs. In cytotoxicity assays, GSK’254 exhibited a mean CC_50_ value of 13 μM (non-PBA), well above the targeted *C*_min_ of 150 nM, supporting GSK’254 application in the clinic and consistent with a first-time-in-human phase I clinical study demonstrating acceptable GSK’254 safety (16). Notably, GSK’254 elicited only mild gastrointestinal intolerability in this clinical study.

GSK’254 also exhibited robust antiviral activity against 8 common HIV-1 laboratory strains and 19 clinical isolates, some containing Gag polymorphisms. Clinical isolates representing HIV-1 subtypes A, B, C, and CRF01_AE were examined, and GSK’254 exhibited strong antiviral activity across this set. Thus, GSK’254 exhibited excellent activity against a broad spectrum of HIV-1 isolates and had an improved antiviral profile compared with the previous MI GSK’795. A prior study found that a compound referred to as BMS MI B exhibited robust antiviral activity toward clinical isolates with Gag/protease genes from participants who failed protease treatment and had emergent protease inhibitor-resistant mutations, (half-maximal fold change effective concentration versus WT [FC-EC_50_] values ≤1.4 [24]). BMS MI B is GSK’254, further supporting the broad-spectrum antiviral activity of GSK’254, even against clinical isolates with protease inhibitor resistance.

GSK’254 was examined against SDMs expressing relevant Gag polymorphisms, with GSK’254 inhibiting most SDMs, and generally displayed activity against genotypes not susceptible to earlier-generation MIs. However, SDMs containing A366V or A364V substitutions exhibited reduced susceptibility to GSK’254. In the most recent version (2019) of the LANL database of HIV-1 sequences, only 0.09% (9 of 10,192 sequences) of genes contained the A364V mutation and there were no instances of A366V (https://www.hiv.lanl.gov/content/index). The A366V substitution, which was identified in earlier bevirimat studies, was reported to induce poor virus replication, inhibit proper virion core condensation, and impart a drug-dependent growth phenotype (25). Drug dependence of A366V-containing viruses is evidenced in Table 5, which shows that these viruses exhibited negative MPI in the multiple-cycle assay format.

The resistance profile of GSK’254 was assessed through breakthrough/selection experiments in cell culture, where virus breakthrough occurred in 9 of 16 samples after 10 days in 80 nM GSK’254 (Table 6). The A364V or A364A/V substitution emerged in 5 of the 9 samples exhibiting breakthrough, with A366V or A366A/V emerging in 4 samples, 1 of which also contained A364A/V. P289P/S was also selected in another sample, but it is unclear what effect this has on MI susceptibility. In another set of selection experiments using increasing concentrations of GSK’254 (Fig. 2), A364V emerged in 4 of 6 cultures, while A224T/A336V/I378M and T332P were selected once each in multipassage assays. The last 2 genotypes were not examined in a site-directed format for susceptibility to GSK’254.

*In vitro*, GSK’254 inhibited the cleavage of p25 to p24 in WT and ΔV370 VLPs (Fig. 3 and Table 7). These results are consistent with the above-described findings showing the antiviral activity of GSK’254 and support the GSK’254 mechanism of action at the MI binding site. However, GSK’254 failed to impact cleavage in the VLPs containing the A364V substitution, consistent with the decreased susceptibility against A364V-containing viruses and the ability to select for this mutation in cells under pressure from GSK’254 under various conditions. Overall, the improved GSK’254 antiviral profile is likely a result of its higher binding affinity and longer residence times once bound to Gag; conversely, its reduced ability to inhibit the replication of the A364V virus and its inability to inhibit *in vitro* cleavage of A364V VLP are likely due to its poorer affinity and extremely rapid dissociation from the A364V binding site.

A series of VLPs with known polymorphisms as well as one with A364V were examined for their rates of cleavage by HIV-1 protease. Interestingly, all the polymorphisms that were identified as impacting the susceptibility of earlier MIs had an increased rate of cleavage compared with that of the WT VLP (Table 7) (22). The fastest of this group was the V362I/V370A-containing VLP, which was cleaved 5.7-fold faster than the WT VLP. GSK’254 remained active against this polymorphism, with V362I/V370A-containing virus having an EC_50_ of 4 nM (MPI, 91%) in the single-cycle assay, an EC_50_ of 3 nM (MPI, 91%) in the multiple-cycle assay (Table 5), and a long dissociative half-life from the V362I/V370A VLP (227 min [Table 7]) and high-affinity binding (0.5 nM [Table 7]). However, VLPs expressing A364V exhibited an innate p25 cleavage rate 9.2-fold greater than that of the WT. This mutation reduced susceptibility to all the MIs (7, 11) and has an extremely short bound residence time. For example, GSK’254 exhibits a dissociative half-life from A364V of ≤1 min as well as 6.5-fold-reduced affinity versus that of the WT (Table 7). Thus, reduced activity toward A364V is correlated with its relatively rapid cleavage of p25 by HIV-1 protease coupled with the substantially reduced MI residence times and poorer affinity to previous- and current-generation MIs. Interestingly, the antiviral ability of GSK’254 did not linearly correlate with the other VLP p25 cleavage rates (Table 7). One possible reason for the lack of correlation is that this assay does not accurately recapitulate the biological process of virus cleavage and maturation that occurs *in vivo*. However, p25 cleavage rates (Fig. 3) broadly correlated with GSK’254 and GSK’795 antiviral activity as assessed in single- and multiple-cycle assays (Table 5). It was previously shown that antiviral activity correlates with p25 cleavage rates using this assay (22), indicating that perhaps other factors beyond binding affinity and the absolute value of the residence time may play roles in susceptibility or there may be a plateau in the effects of these parameters that control susceptibility to MIs.

In individuals receiving GSK’795 monotherapy for 10 days, A364A/V was selected in 6 participants by day 11 (11), and in the phase IIb study in which GSK’795 was used along with tenofovir disoproxil fumarate and emtricitabine for 24 weeks, A364V was present in 1 of 8 participants who met criteria for resistance testing and had Gag genotype sequencing (13). In a phase IIa proof-of-concept study with GSK’254 at 200 mg, A364V emerged in 4 of 6 participants receiving 10-day monotherapy, with 1 of the 4 participants showing phenotypic resistance. However, when the study was shortened to 7 days of dosing, no A364V mutations emerged (17). Altogether, these findings indicate that A364V remains an important substitution to monitor while assessing the clinical utility of the MI GSK’254.

A limitation of this study is that though the panel of tested viruses were highly enriched in Gag variations known to confer reduced susceptibility to previous generations of MIs, this might still represent only a subset of total Gag variations. In any case, the present findings demonstrate robust antiviral properties of GSK’254 against viruses with Gag polymorphisms and viruses selected for resistance to the prior-generation MI GSK’795, except for rare genotypes. Furthermore, GSK’254 demonstrated a 2-log_10_ viral load reduction in a phase IIa proof-of-concept study (17), and phase IIb trials are currently progressing (ClinicalTrials.gov identifiers NCT04493216 and NCT04900038). Overall, these results support the ongoing clinical development of GSK’254 as a next-generation MI for HIV-1 treatment.

## MATERIALS AND METHODS

### Compounds, cell lines, and viruses.

**(i) Compounds.** GSK’254 (formerly BMS-986173) and GSK’795 (formerly BMS-955176) were discovered and synthesized at Bristol Myers Squibb (Wallingford, CT) (10, 26). ^3^[H]-labeled surrogates for GSK’795 and GSK’254 were synthesized at Bristol Myers Squibb by tritiating the C-20/C-30 double bond in each compound (Fig. 1A).

### (ii) Cell lines.

CEM-NKR-CCR5-Luc and MT-2 cell lines were obtained from the National Institutes of Health (NIH) AIDS Research and Reference Reagent Program, and HEK 293T cell lines were obtained from the American Type Culture Collection (Manassas, VA). CEM-NKR-CCR5-Luc, MT-2, and HEK 293T cells were subcultured twice a week in either Roswell Park Memorial Institute (RPMI) 1640 medium (CEM-NKR-CCR5-Luc and MT-2) or Dulbecco’s modified Eagle medium (HEK 293T). Cell lines were supplemented with 10% heat-inactivated fetal bovine serum and 100 units/mL of penicillin G with 100 units/mL of streptomycin. Media-containing CEM-NKR-CCR5-Luc cells were supplemented with 0.8 mg/mL of G418. Dulbecco’s modified Eagle medium was supplemented with 10 mM HEPES, 2 mM l-glutamine, and 0.25 μg/mL of amphotericin B. All cell culture reagents were manufactured by Gibco Laboratories (Gaithersburg, MD).

Peripheral blood mononuclear cells (BioReclamation, Westbury, NY) were cultured at 37°C and 5% CO_2_ in RPMI 1640 medium with 20% fetal bovine serum (Gibco Laboratories), 100 units/mL of penicillin G, 100 units/mL of streptomycin (Gibco Laboratories), and 1 ng/mL of recombinant human interleukin-2 (Invitrogen, Carlsbad, CA). Peripheral blood mononuclear cells were stimulated with 2 μg/mL of phytohemagglutinin (Sigma-Aldrich, St. Louis, MO) for 2 days before infection with HIV-1 clinical isolates.

### (iii) Viruses.

The NL_4-3_ provirus was obtained from the NIH AIDS Research and Reference Reagent Program and modified such that a section of *nef* was replaced with the *Renilla* luciferase gene (NLRepRlucP373S vector) (10). This construct was used to generate polymorphic variants in Gag either by site-directed mutagenesis or through replacement of the *gag/pr* genes with those derived from clinical isolates. Each variant recombinant virus DNA was used to produce virus stocks by transfecting HEK 293T cells using a Lipofectamine PLUS kit (Invitrogen) as described previously (10).

### (iv) Generation of *gag/pr* HIV-1 genotyped viruses.

Recombinant viruses with cloned *gag/pr* sequences were generated by population cloning *gag*/*pr* from clinical isolates (panel of clinical isolates listed in Table S1). Isolates were obtained from the NIH AIDS Research and Reference Reagent Program or from clinical trials conducted by Bristol Myers Squibb (informed consent was obtained). Gag and protease genes of selected viruses were amplified via reverse transcriptase polymerase chain reaction (PCR) with forward primer 5′-GACTCGGCTTGCTGAAGCGCGCACGGCAAGAGGCGAGGGGCGGCG-3′ and reverse primer 5′-CAGGCCCAATTTTTGAAATTTTTCC-3′. The 2-kb *gag/pr* PCR product was run on a 1.5% agarose gel and purified with the MinElute PCR purification kit (Qiagen, Venlo, Netherlands). DNA sequencing was performed in-house and analyzed using Sequencher software (version 5.0; Gene Codes Corporation, Ann Arbor, MI). The *gag/pr* fragments were digested with BssHII/XmaI and ligated into the similarly digested luciferase reporter vector pNLRepRluc. Percentages of the Gag polymorphic variations and any selected substitutions within the worldwide HIV population were derived through analysis of the LANL 2019 HIV sequence database (https://www.hiv.lanl.gov/content/index; accessed 21 May 2021). Viral strains expressing Gag mutations were selected based on previously reported resistance to MIs (7, 10).

### (v) Preparation of HIV-1 VLPs.

Noninfectious HIV-1 VLPs containing only structural proteins were expressed from a synthetic gene under the control of a cytomegalovirus promoter as previously described (10). Two days after transfection into HEK 293T cells, supernatants containing secreted VLPs were cleared from cell debris by filtration (0.45-μm filter; Millipore, Burlington, MA). The VLPs were then pelleted through a 20% sucrose cushion at 25,000 rpm in an SW28 rotor for 2 h, resuspended in phosphate-buffered saline (PBS) to a total protein concentration of ∼1,000 μg/mL, and stored at −80°C. Virus-like particles containing a range of Gag polymorphisms were constructed.

### Drug susceptibility assays.

Multiple-cycle assays using recombinant viruses or clinical isolates were used to assess MI antiviral activity as described previously (10). pNLRepRlucP373S (the P373S change is in the Gag gene and converts an outlier polymorphism in the NL_4-3_ gene to the prevalent amino acid in the population [11]) plasmids containing SDMs in Gag or *gag/pr* regions from clinical isolates were transfected into HEK 293T cells, and the supernatant containing recombinant virus was harvested after 4 to 5 days. Virus was used to infect MT-2 cells in the presence of a 3-fold serially diluted compound at a final dimethyl sulfoxide (DMSO) concentration of 1%. The inocula of the reporter viruses were determined based on the luciferase activity detected on day 4 postinfection. The MOI (ratio of infectious virions to target cells) of reporter-free laboratory strains and clinical isolates was 0.005, as derived from a tissue culture infectious dose study. Clinical isolates or reporter-free viruses were examined in the reporter CEM-NKR-CCR5-Luc cells or PBMCs at MOIs ranging from 0.005 to 0.01. Each infection started with a drug-free preincubation of the cell-virus mixtures, performed at 37°C and 5% CO_2_ for either 1 h at 2 times the final cell density (reporter viruses) or 1 h (reporter-free laboratory strains) or 3 h (clinical isolates) using cell pellets in 0.5 mL of medium. After preincubation, cell-virus mixtures were added to compound-containing 96-well plates for a final cell density of either 10,000 (MT-2 cells for reporter viruses), 20,000 (MT-2 and PM1 cells for reporter-free laboratory strains), 200,000 (PBMCs for clinical isolates), or 500,000 (CEM-NKR-CCR5-Luc for reporter-free laboratory strains) cells per well. The CEM-NKR-CCR5-Luc cell assay was performed in the presence of Polybrene (10 μg/mL). Virus growth was determined on days 4 to 5 postinfection for reporter variants or days 5 to 11 for the other viruses. The reporter viruses were detected by a luciferase assay according to manufacturer protocols (Brite-Glo luciferase kit [Promega] for CEM-NKR-CCR5-Luc cells and Dual-Luciferase Reporter 1000 assay system [Promega] for the other cells), and luminescence was detected on a Wallac Trilux. The growth of clinical isolates in PBMCs was quantitated by reverse transcriptase activity in a 96-well scintillation proximity assay (SPA). Twenty microliters of culture supernatants was mixed with 40 μL of reaction cocktail (62.5 mM Tris-HCl [pH 7.8], 100 mM KCl, 0.0625% NP-40, 2.5 mM dithiothreitol [DTT], 6.25 mM MgCl_2_, 1 mM EGTA, 6.25 μg/mL of poly rA, 2.5 μg/mL of streptavidin SPA beads prebound with biotinylated-dT12-18, and 1 μCi of [^3^H]TTP). Mixtures were incubated at 37°C for ∼3 h and quenched with 100 μl of 0.25 M EDTA per well. Luminescence was detected on a Wallac Trilux programmed for tritium counting with SPA beads in opaque plates. The growth of the reporter-free laboratory M-tropic strains and clinical isolates was monitored by a p24 enzyme-linked immunosorbent assay (ELISA; PerkinElmer Life Sciences, Waltham, MA) performed on culture supernatants. The incubation was terminated when the control infection (no drug) displayed a significant level of p24 antigen (*A*_490_ ≥ 0.6 at 1:1,000 dilution) in cell supernatants (typically 6 to 10 days postinfection). A 1:1,000 dilution of cell supernatant in PBS was generally observed to fall in the dynamic range of the endpoint signal.

The EC_50_ values for all assays were calculated by using the exponential form of the median effect equation where percent inhibition = 100 × {1/[1+ (EC_50_/drug concentration)*^m^*]}, where *m* is a parameter that reflects the slope of the concentration-response curve (27). Maximal percent inhibition values were calculated as MPI = 100 – (mean signals at the 2 highest concentrations of compound/mean signal of 2 no-drug control wells) × 100.

Single-cycle infection assays were performed as described previously (10). Half-maximal effective concentration values were calculated as the compound concentration that inhibits 50% of the maximal signal (no-drug control), correcting for background. Background was determined as the residual signal observed upon inhibition at the highest concentration of a control protease inhibitor, nelfinavir (3 μM). Half-maximal fold change effective concentration versus WT values were calculated by dividing the EC_50_ of the recombinant virus by the EC_50_ of WT RepRlucNL_4-3_ virus run in parallel.

The effect of human serum on the antiviral activity of GSK’254 toward NLRepRlucP373S-based viruses was determined using a drug sensitivity assay in the presence of 10% fetal bovine serum/40% human serum supplemented with 27 mg/mL of human serum albumin for a final serum albumin concentration of approximately 45 mg/mL (normal levels of human serum albumin are 35 to 50 mg/ml). Luciferase activity was used as the endpoint. Protein binding of GSK’254 in human serum was also directly measured via an ultracentrifugation assay as described previously (10).

Cytotoxicity of GSK’254 was assessed in MT-2 cells after a 4-day incubation in the presence of serially diluted compound (28). The assay was performed in a phenol red-free medium at an initial cell density of 10,000 per well in a 96-well plate format. Cell viability was determined using a redox dye assay [3-(4,5-dimethylthiazol-2-yl)-5-(3-carboxymethoxyphenyl)-2-(4-sulfophenyl)-2*H*-tetrazolium, inner salt (MTS); Promega, Madison, WI] according to the manufacturer’s protocol. Half-maximal cytotoxic concentration values were calculated by fitting to a 4-parameter logistic formula *y* = *A* + {[*B* − A]/1 + [(*C*/*x*)*^d^*]}, where *A* and *B* are minimal and maximal percent inhibition, respectively, *C* is the EC_50_, *d* is the Hill slope, and *x* represents compound concentrations.

### Gag p25 cleavage assay.

As previously described (22), VLPs (∼100 ng) were diluted (1:10) in 10 μL of VLP buffer (50 mM MES [pH 6.0], 100 mM NaCl, 2 mM EDTA, and 2 mM dithiothreitol) supplemented with 0.06% Triton X-100 and incubated at room temperature for 10 to 30 min to remove lipid bilayers. Delipidated VLPs were incubated with either GSK’254 or GSK’795 (3 μM in 0.1% dimethyl sulfoxide), or no compound (control), at 22°C for 2 h and then digested at 22°C for up to 4 h with 0.27 μM HIV-1 protease (constructed to contain substitutions that limit autoproteolysis) (29). Samples (1 μL) were taken at 0-, 0.5-, 2-, and 4-h time points and underwent trypsin digestion. Digestion proceeded overnight at 37°C, and reactions were halted with 1 μL of formic acid. Peptide samples were then analyzed by liquid chromatography with mass spectrometry using a nanoAcquity ultraperformance liquid chromatography system (Waters Corporation, Milford, MA) interfaced with an LTQ XL Orbitrap mass spectrometer (Thermo Fisher Scientific, Waltham, MA). Data were acquired using an Advance CaptiveSpray ion source (Michrom Bioresources Inc., Auburn, CA).

Signals for SP1 peptides were normalized to an internal protease fragment at the C-terminal end of Gag, and data were analyzed using Thermo Xcalibur processing software 3.0.63 and Thermo Xcalibur Quanbrowser software 3.0.63 (Thermo Fisher Scientific) as described previously (23).

### Kinetics of MI dissociation from HIV-1 Gag VLPs.

Maturation inhibitor dissociation rates were measured as previously described (22). Briefly, tritium-labeled versions of surrogate molecules of GSK’254 and GSK’795 were prepared by tritiation at the C-20/C-30 double bond of each molecule (Fig. 1A). These reduced molecules exhibited antiviral properties similar to those of the parent compounds. After binding 30 nM ^3^H-MI to SPA bead/VLP complexes, a 100-fold M excess of unlabeled GSK’254 or GSK’795 was added to effect irreversible displacement of the bound ^3^H-MI. Kinetics of disappearance of the bound ^3^H-MI were monitored using a Microbeta2 plate reader (PerkinElmer), and the data were fitted to a first-order exponential equation (GraphPad Prism v5.1).

### Specific binding of MIs to HIV-1 Gag VLPs.

Specific binding of MIs to Gag was demonstrated using an SPA radiolabeled binding assay as previously described (10). In a 96-well white low-binding plate (Corning; Corning, NY), 0.5 to 1.2 μg of WT or other polymorphic or mutant VLPs was mixed in 40 μl of PBS with SPA beads (100 μg/well, PVT-WGA SPA beads; PerkinElmer) for 1 h at room temperature, and then the volume was increased to 200 μl/well by the addition of binding buffer (100 mM Tris [pH 6.5], 2 mM EDTA, 0.03% Tween 20, and 5 mM MgCl_2_). Serial dilutions of ^3^H-MIs were then added to wells. The final concentration of DMSO in the assay was 10% (vol/vol). After an approximately 4-h equilibration at room temperature, bound ^3^H-MIs were measured using a Top Count or Microbeta2 plate reader (PerkinElmer). *K_d_* values were calculated by fitting the data using GraphPad Prism v5.1.

### Virus breakthrough in the presence of compound.

Virus breakthrough in cell culture was assessed in the presence of GSK’254, GSK’795, or a control (no compound). MT-2 cells were infected at an MOI of 0.005 (low MOI) or 0.05 (high MOI) in 80 nM GSK’254 or GSK’795 with variants of RepRlucNL_4-3_GagP373S (WT V370A, V362I, or V370M). Cultures were incubated at 37°C and 5% CO_2_ until ∼100% cytopathic effect (CPE) was observed. Every 3 to 4 days, cultures were transferred to a new well with fresh medium (1:3 ratio of culture to medium) in the presence of a fixed concentration (80 nM) of GSK’254 or GSK’795. At each passage, 100 μL of culture supernatant was collected and stored at −20°C. When a culture reached 100% CPE, it was terminated, and supernatants were harvested for subsequent virus population sequencing.

### Resistance development through sequential passages in increasing concentrations of GSK’254.

Viral GSK’254 resistance was also assessed using a multipassage approach beginning with a low compound concentration (2 nM) that was increased 2-fold with each passage. NL_4-3_GagP373S (WT) and the SDM NL_4-3_ GagP373SV370A (V370A) were used to start the selections. These variants were selected as parent virus based on common WT and V370A expression in HIV-1 subtypes (47% WT and ∼36% V370A as indicated by the LANL HIV sequence database (https://www.hiv.lanl.gov/content/index). Each selection was performed in triplicate, starting with 2 × 10^6^ MT-2 cells infected with each virus at an MOI of 0.005 (50% tissue culture infectious dose determined by CPE and cultured in 2 × 10^5^ cells/mL in compound-containing medium). Each passage was terminated at ∼100% CPE. To passage virus, the cultures were centrifuged, and 25 μL of viral supernatants was transferred into 10 mL of a fresh cell culture in the presence of a 2-fold-increased compound concentration. The remaining virus supernatant was frozen at −80°C for subsequent sequencing. The experiment concluded at passage 11 when cytotoxicity was present (∼1 μM GSK’254; approximately 4 months).

### Data availability.

Anonymized individual participant data and study documents can be requested for further research from https://www.clinicalstudydatarequest.com/.
