# Supplementary material for: GSK3640254 Is a Novel HIV-1 Maturation Inhibitor with an Optimized Virology Profile
Source: Antimicrob Agents Chemother. 2022 Jan 18;66(1):e01876-21. doi: 10.1128/AAC.01876-21 (PMC8765437; doi:10.1128/AAC.01876-21)
Supplement: Supplemental file 1 — Supplemental Table S1. Download AAC.01876-21-s0001.pdf, PDF file, 0.1 MB [file aac.01876-21-s0001.pdf]

SUPPLEMENTAL TABLE

Supplemental Table S1. EC_{50} and PBA EC_{90} Values in Multiple-Cycle Assays of Selected SDMs and Gag/Pr Genotyped HIV-1 Subtype B and C Viruses

			GSK'254		GSK'795	
**Virus panel**	**Virus name or *gag*/*pr* sequence (SDM)**	**Key amino acid position[a]**	**EC_{50} (μM)**	**PBA EC_{90} (μM)**	**EC_{50} (μM)**	**PBA EC_{90} (μM)**
		**HIV-1 subtype B viruses**				
SDM	Wild-type	V, QVT	0.003	0.024	0.005	0.11
	V370A	V, QAT	0.002	0.021	0.006	0.05
	ΔV370	V, QT	0.003	0.057	0.006	1.03
	V362I/V370A	I, QAT	0.003	0.018	≥3	NA
	V370A/ΔT371	V, QAΔ	0.003	0.040	0.007	0.23
	T332S/V362I/PrR41G	I, QVT	0.007	0.077	0.7	422
	A326T/V362I/V370A	I, QAT	0.026	0.117	≥3	NA
	R361K/V362I/L363M[b]	I, QVT	0.003	0.050	0.552	321
	S368R/V370A	V, QAT	0.001	0.009	0.002	0.04
Wild-type[c]	411-1-C	V, QVT	0.0005	0.007	0.001	0.04
	220162	V, QVT	0.002	0.040	0.005	0.6
	28-MDR	V, QVT	0.001	0.037	0.006	0.1
	4NRR	V, QVT	0.001	0.025	0.001	0.07
	92US729	V, QVT	0.001	0.013	0.001	0.07

V362I	93US141	I, QVT	0.002	0.037	0.009	0.25
	92TH026	I, QVT	0.004	0.032	0.010	0.21
	1000156	I, QVT	0.004	0.063	0.009	0.6
	1900140	I, QVT	0.001	0.047	0.003	1.93
	93TH0647	I, QVT	0.002	0.023	0.003	0.06
	92US072	I, QVT	0.001	0.021	0.003	0.13
	92TH014	I, QVT	0.001	0.012	0.005	0.67
	93BR008	I, QVT	0.002	0.030	0.004	0.12
	93BR024	I, QVT	0.007	0.180	0.054	NA
V362I/V370A	1000038	I, QAT	0.003	0.043	1.4	977
V370A	400051	V, QAT	0.001	0.021	0.002	0.03
V370A/ΔV370	93BR022	V, QAΔ	0.003	0.028	0.010	0.63
	93BR017	V, QAΔ	0.002	0.020	0.016	0.6
	92BR018	V, QAΔ	0.001	0.026	0.003	0.97
V370M	400024	V, QMT	0.001	0.010	0.019	79
	92BR014	V, QMT	0.001	0.011	0.004	0.37
	93BR009	V, QMT	0.002	0.018	0.002	0.13
	1900120	V, QMT	0.001	0.011	0.008	6
V370T	93US155	V, QTT	0.001	0.014	0.005	0.1
		**HIV-1 subtype C viruses**				
	1700141	V, QAΔ	0.002	0.019	0.008	0.37
	11657-3	V, QΔT	0.002	0.037	0.006	0.4

10215-6	V, QΔT	0.001	0.018	0.007	12
98CN009	V, QΔT	0.001	0.027	0.011	1.87
1300495	V, QAΔ	0.001	0.019	0.022	2.27
98IN026	V, QAN	0.001	0.016	0.003	0.07
97ZA003	V, QAΔ	0.001	0.024	0.006	2.4
93MW690	V, QAΔ	0.001	0.017	0.010	1.37
20706-3	V, QΔT	0.001	0.023	0.004	0.37
20635-4	V, ΔΔT	0.001	0.040	0.016	1.7
1900543	V, SAS	0.001	0.024	0.007	0.28

EC_{50}, half maximal effective concentration; EC_{90}, 90% effective concentration; GSK'254, GSK3640254; GSK'795, GSK3532795; NA, not available; PBA, protein-binding adjusted; SDM, site-directed mutant. [a]At amino acid positions 362, 369, 370, and 371. [b]Prepared from a resistant virus identified at baseline in a participant from phase IIa proof-of-concept study (1). [c]Wild-type *gag/pr* defined as viruses with no changes to canonical subtype B V362, Q369, V370, T371.

REFERENCES

1. Dicker I, Zhang S, Ray N, Beno BR, Regueiro-Ren A, Joshi S, Cockett M, Krystal M, Lataillade M. 2019. Resistance profile of the HIV-1 maturation inhibitor GSK3532795 in vitro and in a clinical study. PLoS ONE 14:e0224076.
